# Synergistic Effects of Plant Polysaccharides and Probiotics: A Novel Dietary Approach for Parkinson’s Disease Intervention

**DOI:** 10.3390/ph19010157

**Published:** 2026-01-15

**Authors:** Ye Jin, Lu Wang, Ruiting Lin, Jing He, Da Liu, Yang Liu, Yongzhi Deng

**Affiliations:** 1 School of Pharmacy, Changchun University of Chinese Medicine, Changchun 130117, China; jy_ccucm@163.com (Y.J.); 13674401855@163.com (L.W.); lrt9343756@outlook.com (R.L.); 15943431618@163.com (J.H.); liuda@ccucm.edu.cn (D.L.); 2 Northeast Asian Institute of Traditional Chinese Medicine, Changchun University of Chinese Medicine, Changchun 130117, China; 3 Public Laboratory Centre, Changchun University of Chinese Medicine, Changchun 130117, China; 4 College of Traditional Chinese Medicine, Changchun University of Chinese Medicine, Changchun 130117, China

**Keywords:** Parkinson’s disease, microbiota-gut-brain axis, gut microbiota, probiotic, plant polysaccharides

## Abstract

Parkinson’s disease (PD), the second most common neurodegenerative disorder globally, relies primarily on dopamine replacement therapy for conventional treatment. This approach fails to reverse core pathological processes and is associated with long-term side effects. Recent research on the microbiota-gut-brain axis (MGBA) has revealed that PD pathology may originate in the gut, forming a vicious cycle from the gut to brain through α-synuclein propagation, gut dysbiosis, intestinal barrier disruption, and neuroinflammation. This offers a novel perspective for managing PD through dietary interventions that modulate the gut microbiome. However, single probiotic or prebiotic interventions show limited efficacy. This review systematically introduces the novel concept of “synbiotics combining medicinal plant polysaccharides with probiotics,” aiming to integrate traditional “medicinal food” wisdom with modern microbiome science. The article systematically elucidates the pathological mechanisms of MGBA dysfunction in PD and the intervention mechanisms of probiotics and emphasizes the structural and functional advantages of medicinal plant polysaccharide as superior prebiotics. The core section delves into the multifaceted synergistic mechanisms between these two components: enhancing probiotic colonization and vitality, optimizing microbial metabolic output, synergistically reinforcing the intestinal and blood-brain barriers, and jointly regulating immune and neuroinflammation. This approach targets the MGBA to achieve multi-level intervention for PD.

## 1. Introduction

Parkinson’s disease (PD), as the second most prevalent neurodegenerative disorder globally, is experiencing a sharp rise in incidence alongside population aging, posing an increasingly severe public health challenge [1]. Currently, drug treatment regimens centered on levodopa primarily function through dopamine replacement and supplementation. While they can alleviate motor symptoms to a certain extent, they cannot reverse or slow the core pathological process of progressive loss of dopaminergic neurons in the substantia nigra [2]. More challenging is that long-term medication often comes with unavoidable side effects such as fluctuating efficacy and dyskinesia, severely limiting patients’ long-term quality of life [3]. Therefore, moving beyond traditional “symptom-based” treatment models and developing novel therapeutic strategies capable of intervening at critical early stages of disease progression to achieve true “disease modification” has become an urgent scientific challenge in this field. In recent years, in-depth research on the microbiome-gut-brain axis (MGBA) has provided a revolutionary perspective for understanding the origin and progression of PD. The Braak hypothesis proposes that the pathology of Parkinson’s Disease may originate in the enteric nervous system, with misfolded α-synuclein subsequently propagating upward along the vagus nerve to the central nervous system, ultimately reaching the substantia nigra of the brain [4]. This hypothesis is strongly supported by clinical observations: up to 80% of PD patients experience gastrointestinal dysfunction, such as chronic constipation, years or even decades before the onset of motor symptoms [5]. Modern research has further revealed that PD patients commonly exhibit characteristic gut microbiota dysbiosis, characterized by a decrease in the abundance of beneficial bacteria producing short-chain fatty acids and an increase in the proportion of pro-inflammatory bacterial communities (such as Enterobacteriaceae) [6]. This dysbiosis can disrupt tight junctions in the intestinal epithelium, leading to “leaky gut”. This allows bacterial toxins such as lipopolysaccharides to enter the circulatory system, activating systemic and central nervous system inflammation, ultimately accelerating the degeneration and death of dopaminergic neurons [7]. The MGBA has now established a robust link between gut microbiota, intestinal barrier function, neuroinflammation, and α-synuclein pathology. Given the MGBA’s pivotal role, dietary interventions targeting gut microbiome regulation represent a highly promising strategy for Parkinson’s Disease management. However, single probiotic or prebiotic interventions have demonstrated inconsistent outcomes in clinical studies, with their limitations becoming increasingly apparent: the efficacy of prebiotics is highly dependent on the host’s existing microbial baseline, while exogenously supplemented probiotics face challenges in colonizing the complex intestinal environment [8]. To overcome this bottleneck, the international food industry introduced the concept of synbiotics. According to the International Scientific Association for Probiotics and Prebiotics (ISAPP), synbiotics are defined as “a mixture of live microorganisms (probiotics) and a substrate (prebiotic) that can be selectively utilized by the host microorganisms, conferring health benefits to the host [9].” Its core concept lies in achieving a synergistic effect where “1 + 1 > 2”: prebiotics provide dedicated “fuel” for the probiotics being supplemented, enhancing their survival and colonization capabilities, thereby enabling stronger and more sustained steady-state regulation of the MGBA.

Traditional Chinese medicine, rooted in the wisdom of “medicinal and edible substances sharing the same origin,” offers us a vast treasure trove. Among these, medicinal plant polysaccharides—a class of macromolecular substances characterized by diverse structures and broad biological activities—are demonstrating tremendous potential as a new generation of prebiotics [10]. Compared to traditional prebiotics such as inulin and fructooligosaccharides, plant polysaccharides—including *Astragalus* polysaccharides, *Lycium* polysaccharides, and *Ganoderma lucidum* polysaccharides—possess unparalleled structural complexity and multifunctionality [11]. This “killing multiple birds with one stone” characteristic makes medicinal plant polysaccharides an ideal “essence” ingredient for constructing highly effective, multifunctional synbiotic formulations, with the potential to generate unprecedented synergistic effects when combined with specific probiotics. This review aims to systematically introduce and substantiate the innovative concept of “medicinal plant polysaccharides-probiotics” for the first time, while thoroughly exploring its scientific rationale and application prospects as a novel dietary approach targeting the MGBA to intervene in PD. This paper will first systematically dissect the core pathological mechanisms of MGBA dysfunction in PD. It will then elaborate on the functional characteristics of probiotics, followed by a detailed exposition of the material basis and functional properties of medicinal plant polysaccharides as exceptional prebiotics. Subsequently, the focus will shift to the synergistic mechanisms between medicinal plant polysaccharides and probiotics, examining multiple dimensions including enhanced probiotic colonization, optimized microbial metabolic outputs (e.g., short-chain fatty acids), joint reinforcement of the intestinal barrier, and collaborative regulation of immune-neuroinflammation. By integrating traditional “medicine-food homogeneity” wisdom with cutting-edge modern microbiome and neuroscience research, this work provides groundbreaking insights and robust theoretical foundations for developing next-generation precision functional foods for PD nutritional management.

## 2. The Relationship Between MGBA and PD

### 2.1. Operational Mechanism of MGBA

**Figure 1:**
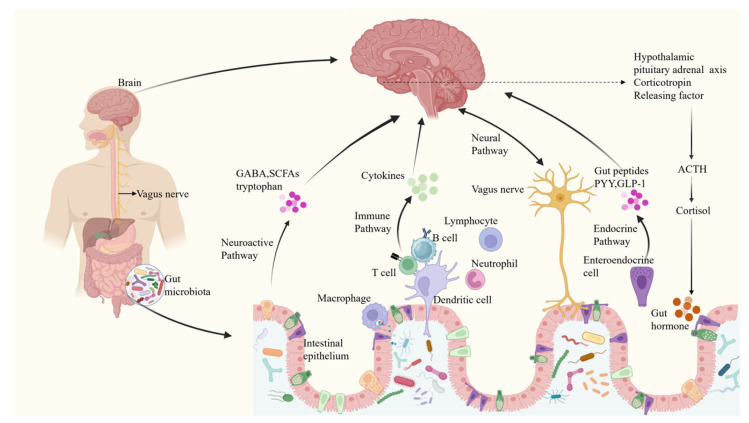
Schematic representation of the mechanism of action of the microbiota-gut-brain axis (MGBA). This figure shows the communication mechanism between the gut and the brain: the gut microbiota communicates with the brain through neural pathways (vagus nerve), neuroactive pathways such as gamma-aminobutyric acid (GABA), short-chain fatty acids (SCFAs), tryptophan, etc., immune pathways such as macrophages, T cells, B cells, lymphocytes, neutrophils and many other immune cells, and endocrine pathways such as gut peptides (PYY), gut hormones, glucagon-like peptide-1 (GLP-1), and also shows that the hypothalamic-pituitary-adrenal axis is involved in this communication. The figure was created in BioRender. Jin, Y. (2026) (https://BioRender.com/xlc7gyt) and the text was created in PowerPoint.

The microbial-gut-brain axis (MGBA) plays a pivotal role in health and in numerous diseases, including neuropsychiatric disorders. Research in this field is being conducted extensively and rapidly. The MGBA refers to a complex bidirectional communication system between gut microbiota and the brain, primarily exchanging information through multiple pathways including neural, neuroactive molecules, immune, and endocrine systems (Figure 1).

#### 2.1.1. Neural Pathway

As the most critical neural pathway of the MGBA, vagus nerve has far more functions than simple signal transduction. It accurately and quickly maps the dynamic state of gut microbiota to the central nervous system by integrating multimodal sensory information from intestinal tract [12]. The most classic is its perception of gut chemistry and hormone signals. The neuromodulators released by intestinal endocrine cells (EECs) in response to gut microbiota metabolites, such as short chain fatty acids, such as cholecystokinin (CCK) and 5-hydroxytryptamine (5-HT), directly activate the corresponding receptors on the afferent terminals of the vagus nerve [13]. The ability of vagotomy to block many central behavioral effects triggered by gut microbiota interventions (such as probiotic intake) provides the most critical causal evidence for this chemical sensing pathway [14]. In addition, the vagus nerve can also be activated by intestinal immunity and pathogen invasion. For example, pathogens such as *Campylobacter jejuni* induce the intestinal tract to produce pro-inflammatory cytokines, which themselves act as signaling molecules that directly or indirectly stimulate the afferent fibers of the vagus nerve, thereby quickly reporting intestinal inflammation or infection to the nucleus tractus solitariae (NTS) of the brain stem and triggering corresponding disease behaviors (e.g., anorexia, anxiety) [15]. Crucially, the newly discovered mechanism of “neuropodocytes” has fundamentally revolutionized our understanding of the speed and precision of gut-brain signaling. These specialized EECs can form true synaptic connections with the afferent terminals of the vagus nerve and use glutamate as a neurotransmitter for signaling [16]. This finding elevates the time scale of the MGBA from minute hormone diffusion to millisecond synaptic transmission, providing a subversive anatomical basis for understanding how intestinal contents instantaneously affect brain function and decision-making (such as feeding behavior and emotional valence). Importantly, the vagus nerve constitutes a bidirectional feedback loop. While its afferent fibers relay gut-derived signals to the brainstem, its efferent fibers convey central commands back to the enteric nervous system and gut wall. This efferent output can modulate gastrointestinal motility, secretion, and local immune responses, thereby allowing the brain to dynamically regulate the gut environment in response to the incoming signals [17]. This closed-loop communication is fundamental to the homeostatic function of the MGBA.

#### 2.1.2. Neuroactive Molecular Pathway

As a huge “ biochemical plant”, the most direct influence of intestinal gut microbiota is to synthesize or induce the host to produce a series of neuroactive molecules (Table 1), which can enter the circulatory system or interact directly with the nervous system through specific pathways, thus remotely regulating brain function and behavior [18]. The most typical and widely studied is the direct contribution of gut microbiota to major neurotransmitter systems. For example, some lactic acid bacteria and bifidobacteria can synthesize gamma-aminobutyric acid (GABA) in situ in the intestinal tract, while *Escherichia coli* and yeast can produce precursors of catecholamines (such as dopamine and norepinephrine [19]. *Bacteroides fragilis* has been shown to significantly increase serotonin (5-HT) levels in the host colon and brain, which are closely related to social behavior, through its unique polysaccharide A (PSA) signaling [20]. Further, these gut microbiota derived neuroactive molecules have been shown to cross the gut brain barrier and trigger definite central physiological and behavioral changes [21]. Gut microbiota metabolites in the circulatory system, such as GABA produced by lactic acid bacteria, have been experimentally confirmed to cross the blood brain barrier and accumulate in the limbic system of the brain, directly activating GABA receptors on specific neurons, and finally exerting similar anti-anxiety effects to diazepam [22]. Importantly, the causal association between gut microbiota metabolome and host behavioral phenotype has been strongly confirmed by the study of sterile animals and fecal bacteria transplantation. Transplantation of intestinal flora from anxiety-like phenotype mice into sterile mice induced similar behavioral abnormalities in the latter, accompanied by a significant down-regulation of brain-derived neurotrophic factor (BDNF) expression in brain regions such as the hippocampus; Conversely, transplanting healthy flora can alleviate this symptom [23,24].

#### 2.1.3. Immune Pathways

The gut microbiota and its metabolites serve as key peripheral factors regulating the immune homeostasis of the central nervous system, with the intestinal mucosal immune system playing a pivotal role in this process. For example, microbiota-specific induced regulatory T cells play a central role in maintaining immune tolerance in the gut and brain [49]. Specifically, commensal bacteria such as *Clostridium* in the gut promote the differentiation and function of local intestinal Tregs by producing short-chain fatty acids (e.g., butyrate). These Treg cells subsequently enter the systemic circulation and migrate to the brain, where they suppress excessive activation of microglia by secreting anti-inflammatory factors IL-10 and TGF-β, thereby alleviating neuroinflammation [50]. Conversely, under dysbiotic conditions, the induction and function of Treg cells are impaired, leading to insufficient immunosuppression and potentially exacerbating the severity of autoimmune diseases such as multiple sclerosis [51]. Beyond Treg cells, other immune cell subsets are also deeply involved. When the gut microbiota activates a subset of IFNγ NK cells, these cells migrate to the central nervous system and induce the production of anti-inflammatory astrocytes. These stellate glial cells induce T cell apoptosis via tumor necrosis factor-related apoptosis-inducing ligand decoy receptor 5 (TRAIL-DR5) signaling, thereby suppressing neuroinflammation [52]. In addition to IFNγ NK cells, meningeal IgA plasma cells that are absent or reduced in germ-free (GF) mice have also been confirmed through B-cell receptor sequencing to originate from the gut. This type of plasma cell can migrate from the gut to the central nervous system, particularly the meninges. Once within the meninges, these IgA plasma cells protect the developing brain from infection by trapping pathogens within the dural sinuses, thereby preventing their entry. This highlights the role of the gut microbiota in preventing infectious encephalitis caused by pathogens by training B-cell immunity [53]. Additionally, the gut microbiota can also shape the phenotypes of other immune cells within the intestinal microenvironment. A classic example is segmented filamentous bacteria, which induce Th17 cell production and migrate to the central nervous system; Under homeostatic conditions, IL-17 secreted by Th17 cells helps maintain blood-brain barrier integrity and defend against pathogen invasion [54]. However, intriguingly, in genetically susceptible individuals or under persistent inflammatory stimulation, these cells may also switch to a pathogenic phenotype, massively producing IL-17 and GM-CSF, thereby driving neuroinflammation and autoimmune damage. In the experimental autoimmune encephalomyelitis (EAE) mouse model, both SLAMF6 stem cell-like Th17 cells—regulated by the gut microbiota to maintain homeostasis—and CXCR6-expressing Th17 cells capable of migrating to the central nervous system to induce neuroinflammation are present [55].

#### 2.1.4. Endocrine Pathways

The gut microbiota constitutes a systemic hormonal pathway influencing neurodevelopment and behavior by precisely regulating the enteroendocrine system and downstream higher neuroendocrine axes. At the local intestinal interface, microbial metabolites directly serve as key regulatory signals for enteroendocrine cells. For example, taurine produced by symbiotic bacteria can promote the synthesis and release of serotonin by activating G protein-coupled receptors on enteroendocrine cells [56]. Conversely, the pathogen-associated metabolite 4-ethylphenyl sulfate negatively regulates intestinal 5-HT levels by depleting tryptophan precursors [57]. Furthermore, these gut-derived hormonal signals are efficiently transmitted to the central nervous system via dedicated neural pathways. Recent studies reveal that the neurites extended by enteric neurons form direct synaptic connections with vagal nerve terminals within the intestine. This enables the conversion of local 5-HT chemical signals into neural impulses within milliseconds, facilitating rapid gut-brain communication [58]. Ultimately, these transmitted signals converge at higher-order integration centers such as the hypothalamus, where they systematically regulate two key neuroendocrine axes, thereby governing macro-level social behaviors and stress responses. In the social behavior pathway mediated by oxytocin, *Lactobacillus reuteri* may promote the release of oxytocin in the paraventricular nucleus of the hypothalamus by activating the aforementioned vagal pathway. This, in turn, modulates the dopaminergic circuitry in the ventral tegmental area, thereby improving deficits in social interaction [59]. Meanwhile, within stress response pathways, the healthy colonization of gut microbiota during early life is crucial for the normal establishment of the hypothalamic-pituitary-adrenal axis. Research indicates that commensal bacteria such as *Enterococcus faecalis* can mitigate early-life stress through epigenetic mechanisms, preventing excessive HPA axis activation and associated anxiety-like behaviors. Conversely, dysbiosis disrupts this axis’s function, leaving individuals persistently vulnerable to stress sensitivity [60]. Mechanistically, the microbiota may influence corticotropin-releasing factor release by modulating the expression of receptors such as NMDA and 5-HT1A in the hypothalamus, thereby calibrating the HPA axis stress response [61].

### 2.2. Dysregulation of the MGBA in PD

Parkinson’s disease (PD) is characterized by abnormal aggregation of α-synuclein (α-syn) affecting all levels of the mind-gut-brain axis (MGBA). This abnormal aggregation disrupts bidirectional communication between the brain and gut, and MGBA dysfunction is considered a key driver in the onset and progression of PD.

#### 2.2.1. Characteristic Gut Microbiota Dysbiosis in PD: Initiating Factors of MGBA Dysbiosis

##### Changes in Gut Microbiota Community Structure in PD

Alterations in gut microbiota structure in PD represent the initiating phase of MGBA dysregulation [62]. Numerous studies have confirmed that, compared to healthy individuals, the relative abundance of gut microbiota with anti-inflammatory and short-chain fatty acid-producing functions (such as *Prevotella* spp.) is significantly reduced in PD patients [63]; meanwhile, bacterial communities with pro-inflammatory potential (such as the *Enterobacteriaceae* family) show relative increases, constituting the core microbial community characteristics associated with PD [63]. Despite heterogeneity among PD patients across different studies, metagenomic analyses and cohort studies of untreated patients strongly indicate that this dysbiosis is an intrinsic feature of PD itself, rather than merely a consequence of medication or comorbid conditions [64]. Furthermore, this ecological imbalance exerts direct and multifaceted functional effects on the pathophysiology of PD. The most direct evidence comes from functional studies: transplanting fecal microbiota from PD patients into α-synuclein-overexpressing mouse models significantly exacerbates their motor deficits, central nervous system inflammation, and pathological α-synuclein aggregation. Conversely, transplanting microbiota from healthy donors produces a protective effect [65], this establishes a causal relationship between dysbiosis and the pathology of PD. Furthermore, structural alterations in the gut microbiota are closely associated with the clinical progression of Parkinson’s Disease. Longitudinal follow-up studies reveal that persistent reduction in specific bacterial genera—such as *Prevotella*—is significantly correlated with accelerated deterioration of motor symptoms and increased non-motor burden in patients [66], microbiome dynamics may serve as biomarkers for predicting disease progression. Ultimately, at the molecular level, specific microbial members can initiate core pathology through direct molecular mimicry. For example, certain strains of *Escherichia coli* can produce bacterial amyloid fibers called Curli proteins, which directly cross-promote the misfolding and fibrillation of human α-synuclein. Within the enteric nervous system, these proteins act as “seeds” for pathological aggregation, potentially serving as molecular initiators supporting the Braak hypothesis that pathology originates in the gut [67].

##### Changes in Metabolic Products of Gut Microorganisms in PD

Among the complex changes in the microbial metabolome, short-chain fatty acids—primarily butyrate, propionate, and acetate—are microbial metabolites and bacterial fermentation products of dietary fiber produced in the colon [67]. As the primary energy source for intestinal epithelial cells, most short-chain fatty acids in the gut are absorbed by colonic cells. Only a small fraction enters the systemic circulation and crosses the blood-brain barrier to regulate systemic immune responses and microglial proliferation. Numerous clinical observations indicate that reduced SCFAs correlate with PD pathology. With the reduction in SCFA-producing microbiota such as *Clostridium difficile* in PD patients, levels of SCFAs including acetate, propionate, and butyrate in their stool are generally significantly decreased. This reduction is significantly associated with more severe bradykinesia, postural instability, and cognitive decline in patients [68]. However, interestingly, when the research focus shifted from intestinal contents to systemic circulation, a contradictory phenomenon emerged: despite reduced SCFA levels in feces, elevated SCFA concentrations were observed in both plasma and saliva of PD patients [69]. This seemingly contradictory phenomenon may find a unified explanation in the core intestinal pathology of PD—intestinal barrier dysfunction (i.e., “leaky gut”) [70]. Increased intestinal permeability may allow SCFAs, which are normally confined to the intestinal lumen and exert their effects locally, to abnormally enter the portal venous circulation in large quantities, leading to an abnormal elevation of their concentration in peripheral blood. This “positional misalignment” is directly linked to the functional shift in SCFAs. Experimental evidence indicates that in transgenic mouse models of Parkinson’s Disease, oral supplementation with SCFAs instead induces microglial activation, exacerbates neuroinflammation, and significantly worsens motor impairment. This strongly suggests that excess SCFAs in the systemic circulation may exert harmful effects on disease progression by activating specific G protein-coupled receptors (such as GPR41 and GPR43) [71]. However, another set of studies in rodent models without PD or with different pathological backgrounds reached entirely opposite conclusions, finding that SCFA treatment enhances the survival rate of dopaminergic neurons by suppressing neuroinflammation, maintaining mitochondrial function, and elevating brain-derived neurotrophic factor (BDNF) levels. Therefore, the effects of SCFAs on the PD brain are highly dependent on their microenvironmental concentration, site of action, and the host’s disease state [72]. In the intestinal tract, the absence of its physiological concentration impairs its beneficial functions, including maintenance of the intestinal barrier and anti-inflammatory regulation of immune cells. However, when abnormally large quantities enter the systemic circulation, they may “turn from friend to foe,” transforming into drivers that promote neuroinflammation.

#### 2.2.2. Intestinal Barrier Dysfunction in PD: A Core Driver of MGBA Dysregulation

One of the most direct consequences of gut dysbiosis is the disruption of the intestinal barrier function, commonly known as “leaky gut” [73]. A healthy intestinal barrier relies on the tight seal formed by tight junction proteins (such as Occludin, ZO-1, and Claudin) between intestinal epithelial cells. In PD, the integrity of this barrier is severely compromised. Researchers found that serum zonulin levels were significantly elevated in PD patients compared to healthy controls. Zonulin, a key physiological protein regulating intestinal tight junction permeability, exhibits elevated levels as a classic marker of “leaky gut” [74]. Subsequent histopathological studies further confirmed this structurally. Analysis of colon biopsy specimens from PD patients by Schwiertz et al. revealed severe disruption in the distribution of the epithelial tight junction protein Occludin. Immunofluorescence staining exhibited a discontinuous, fragmented abnormal pattern, visually demonstrating structural damage to the physical barrier [75]. This barrier disruption mechanism is twofold. On one hand, there is insufficient production of SCFAs (particularly butyrate) by beneficial bacteria. Butyrate serves as the primary energy source for colonic cells and is crucial for maintaining tight junctions. The negative correlation between butyrate concentrations in PD patients’ feces and intestinal permeability markers confirms the absence of its protective effect [76]. On the other hand, potentially harmful bacteria and their metabolites (such as LPS) directly attack the body. The Devos team’s report indicates that LPS-binding protein levels are significantly elevated in the serum of PD patients, with concentrations positively correlated with disease severity and inflammatory markers. This directly demonstrates the translocation of bacterial toxins from the gut into the systemic circulation [77]. This study further demonstrated in cellular models that serum from PD patients can induce activation of human brain microvascular endothelial cells, an effect blocked by inhibitors of the LPS receptor TLR4. This clearly delineates the causal chain linking “intestinal leakage” to systemic inflammation. Once LPS enters the circulation, it acts as a potent inflammatory trigger, binding to Toll-like receptor 4on immune cells such as macrophages and activating core inflammatory signaling pathways like NF-κB. Research has confirmed that PD patients exhibit upregulation of TLR4 expression in peripheral blood mononuclear cells, accompanied by sustained activation of the NF-κB pathway [78]. This leads to a storm-like release of downstream pro-inflammatory factors: multiple independent studies have consistently found significantly higher levels of tumor necrosis factor-α, interleukin-1β, and interleukin-6 in the plasma or serum of PD patients compared to healthy controls [79].

#### 2.2.3. Pathological Signal Transmission: The Causal Bridge of MGBA Dysregulation

Experimental evidence supporting the Braak hypothesis has steadily accumulated in recent years. Kim et al. found that injecting small amounts of human pathological α-synuclein preformed fibrils into the duodenal wall of mice. The results clearly demonstrated that misfolded α-syn first appeared in the myenteric plexus of the intestine, then ascended specifically along the vagus nerve, sequentially reaching the dorsal nucleus of the vagus nerve, the locus coeruleus, and ultimately the substantia nigra. This propagation process was completely blocked following vagotomy [80]. This provides direct experimental evidence for the “prion-like,” trans-synaptic propagation of α-syn. At the human epidemiological level, studies have found that individuals who underwent vagotomy for peptic ulcer disease exhibited a significantly reduced risk of subsequent PD. This reduction was particularly pronounced in patients who underwent surgery at least five years prior to PD onset [81]. This finding strongly suggests that severing the “neural highway” connecting the gut to the brain can significantly reduce the risk of PD onset, providing compelling population-based evidence for the vagus nerve’s pivotal role in the pathological spread of PD in humans. Systemic low-grade inflammation triggered by “leaky gut” fills the bloodstream with pro-inflammatory cytokines, which penetrate and affect the central nervous system through multiple mechanisms. First, these inflammatory signals can breach the brain’s defenses. Research by Borghammer et al. confirmed that pro-inflammatory factors (such as TNF-α) in the serum of PD patients can activate cerebral vascular endothelial cells. By downregulating the expression of tight junction proteins, they increase the permeability of the blood-brain barrier, thereby opening pathways for more peripheral inflammatory factors to enter the brain parenchyma [81]. Once these signals enter the central nervous system, they strongly activate microglia. In vitro studies have demonstrated for the first time that stimulating primary microglia with serum from PD patients or MPTP-treated mouse models induces polarization toward the pro-inflammatory M1 phenotype, accompanied by substantial release of TNF-α, IL-1β, and reactive oxygen species. This activation effect is significantly suppressed upon administration of TLR2/4 receptor antagonists. In vivo studies further validated this finding [82]. Pott Godoy et al. successfully induced delayed, progressive dopaminergic neuron-specific loss accompanied by sustained microglial activation by continuously infusing low-dose LPS into the rat substantia nigra (mimicking systemic inflammation). This perfectly replicates the key features of chronic neuroinflammation and selective neuronal damage characteristic of PD [83].

#### 2.2.4. Reception and Amplification of Central Signals: The Final Manifestation of Parkinson’s Disease

When pathological signals originating from the gut ultimately reach the substantia nigra, a self-perpetuating vicious cycle is initiated and rapidly amplified within the brain, ultimately culminating in irreversible neuronal damage. Microglia-mediated chronic neuroinflammation serves as the central driving force in this process. Numerous postmortem studies have identified evidence of sustained microglial activation in the brains of Parkinson’s Disease patients. The study by Croisier et al. demonstrated a significant increase in the density of activated microglia in the substantia nigra of Parkinson’s Disease patients [84]. This density was negatively correlated with the number of remaining dopaminergic neurons, providing direct morphological evidence of a close spatial and temporal association between these two factors throughout the disease progression. These activated microglia are not benign; they release a significant volume of neurotoxic substances. Both initial and subsequent studies have confirmed that microglia isolated from the brain tissue of Parkinson’s Disease patients secrete elevated levels of tumor necrosis factor-α (TNF-α) and interleukin-1β (IL-1β) in their culture supernatants [85]. TNF-α can directly initiate programmed cell death in dopaminergic neurons by activating signaling pathways downstream of its receptors, such as the caspase-8 pathway. Crucially, activated microglia also generate substantial extracellular reactive oxygen species (ROS) and reactive nitrogen species (RNS) [86]. These external oxidants directly deplete the limited antioxidant capacity of adjacent dopaminergic neurons and inhibit the nuclear translocation of the key antioxidant transcription factor Nrf2 within neurons, thereby suppressing the expression of endogenous defense genes. The mitochondria of dopaminergic neurons are particularly susceptible. As noted by Flores-Ponce et al., dopaminergic neurons in the substantia nigra exhibit exceptionally high energy demands and oxidative metabolic activity, rendering them more vulnerable to pathological insults [87]. When dopaminergic neurons face disruption of their redox homeostasis, these neurons become abnormally vulnerable due to elevated baseline oxidative stress resulting from dopamine metabolism, increased iron content, and heightened calcium oscillations [88]. Indeed, inflammatory cytokines such as IL-1β have been demonstrated to impair the activity of mitochondrial complex I. This damage leads to increased electron leakage in the respiratory chain, generating superoxide anions and initiating a cascade reaction of mitochondrial ROS production [89]. This mechanism is fully consistent with the mitochondrial dysfunction observed in Parkinson’s Disease (PD), thereby aggravating cellular energy crisis and oxidative stress [90]. Pathological α-synuclein (α-syn) is an intrinsic cause of mitochondrial dysfunction and oxidative stress. Emerging evidence indicates that oligomerized α-syn can translocate into mitochondria via the TOM20/TOM40 import complex. Once internalized, it accumulates and directly inhibits Complex I function, further exacerbating ROS production at the source. Furthermore, α-syn aggregation at presynaptic terminals disrupts synaptic vesicle recycling, leading to abnormal cytoplasmic dopamine accumulation and autooxidation. This process generates reactive dopamine quinone and hydrogen peroxide, constituting another major source of intracellular oxidative stress within neurons [91,92]. Products of oxidative stress, such as reactive oxygen species (ROS), can in turn further activate the NLRP3 inflammasome, thereby promoting the production and release of additional pro-inflammatory cytokines, including IL-1β [93]. This forms a fatal triangular vicious cycle that continuously self-amplifies. Under such conditions, pathological alpha-synuclein (α-syn) acts as an “accelerator”. Co-culturing recombinant α-syn with microglia potently induces the production of nitric oxide and superoxide in microglia, a process dependent on the TLR2 receptor expressed on the microglial surface [94]. This indicates that pathological α-syn transmitted via the vagus nerve is not merely a passive bystander. Once it enters the centrally inflamed environment, which has already been pre-activated by inflammation, it further potently stimulates microglia to produce an increased amount of neurotoxic substances. Ultimately, this self-sustaining destructive network—composed of chronic microglial activation, cytokine storms, oxidative stress, mitochondrial failure, and the spread of pathological α-syn—exceeds the self-repair threshold of dopaminergic neurons, leading to their progressive and irreversible loss [94]. When the loss of dopaminergic neurons in the substantia nigra reaches 50–60%, accompanied by an approximately 80% reduction in striatal dopamine levels, the characteristic clinical motor symptoms (tremor, rigidity, and bradykinesia) become evident [95]. This functionally confirms that the central reception and amplification process serves as the final pathway through which Parkinson’s Disease progresses from a concealed intestinal origin to overt neurological dysfunction. Furthermore, the heterogeneity of PD itself—manifested in distinct clinical subtypes—also plays a crucial role in shaping MGBA dysfunction and influencing the efficacy of microbiome-targeted interventions.

### 2.3. Parkinson’s Disease Subtypes and Their Impact on MGBA Dysregulation

PD is not a monolithic disorder but a syndrome with diverse clinical and pathological subtypes, which may differentially affect MGBA integrity and response to therapeutic strategies. Parkinson’s Disease (PD) is not a single disease entity but a complex syndrome exhibiting high heterogeneity in clinical symptoms, pathological progression, biochemical alterations, and genetic background (Table 2). This heterogeneity directly impacts the efficacy of any intervention strategy, including Synthroids [96]. The classification of PD subtypes has evolved from a purely clinical phenotype to a multidimensional system integrating biomarkers. Based on predominant symptoms, it is primarily divided into two categories: motor subtypes—tremor-dominant, postural instability/gait disorder, and mixed [97]. Non-motor subtypes: include rapid eye movement sleep behavior disorder (RBD)-predominant, cognitive impairment (e.g., mild cognitive impairment)-predominant, autonomic dysfunction (e.g., severe constipation, orthostatic hypotension)-predominant, and depression/anxiety-predominant [98]. Research has found that after 12 weeks of probiotic supplementation in patients with TD-type PD, not only did constipation symptoms show significant improvement (as assessed by the Constipation Rating Scale), but the amplitude of resting tremor (measured using wearable accelerometers) also exhibited a decreasing trend [99]. Researchers speculate that this may be related to probiotics modulating gamma-aminobutyric acid (GABA) or glutamatergic neurotransmission within the thalamocortical circuits associated with tremor generation. This modulation may originate from the regulation of vagal afferents or systemic immunity by gut microbiota metabolites, such as short-chain fatty acids. The TD subtype is considered to have a relatively benign disease course and involve fewer pathologies beyond dopaminergic neuron loss. Synbiotic formulations targeting this subtype may focus on improving intestinal motility and reducing local oxidative stress, thereby potentially stabilizing the influence of peripheral nerve activity on central motor circuits. For the PIGD/cognitive impairment subtype, synbiotic formulations should be specifically targeted toward “neuroprotection” and “anti-inflammation”. For example, combining probiotics capable of strongly stimulating butyrate production with medicinal plant polysaccharides possessing systemic anti-inflammatory effects may synergistically downregulate neuroinflammation possessing systemic anti-inflammatory effects may synergistically downregulate neuroinflammation, thereby providing a more favorable microenvironment for dopaminergic neurons and cognition-related brain regions. For RBD or PD patients exhibiting RBD features, synbiotics should aim beyond symptom management to target α-synuclein aggregation and propagation [100]. Research indicates that certain probiotics can produce enzymes capable of degrading α-synuclein oligomers, while some plant polysaccharides have been demonstrated to inhibit the misfolding and aggregation of α-synuclein. Combining these elements may constitute an entirely novel “neuropathology-targeted” synbiotic. Here, the role of medicinal plant polysaccharides as prebiotics is crucial [101,102]. For example, dietary fibers such as konjac glucomannan and inulin have been proven to effectively promote intestinal motility and increase stool bulk. Combining them with probiotics that metabolize these polysaccharides to produce gas and short-chain fatty acids—further stimulating intestinal peristalsis, creating a potent “motility-promoting synbiotic” that specifically alleviates this core symptom [103,104]. In summary, the clinical and pathological subtype differences in PD profoundly influence the dysregulation patterns of the gut-brain axis, thereby determining the efficacy of microbial intervention strategies represented by synbiotics.

## 3. The Effects of Probiotics on Parkinson’s Disease

**Figure 2:**
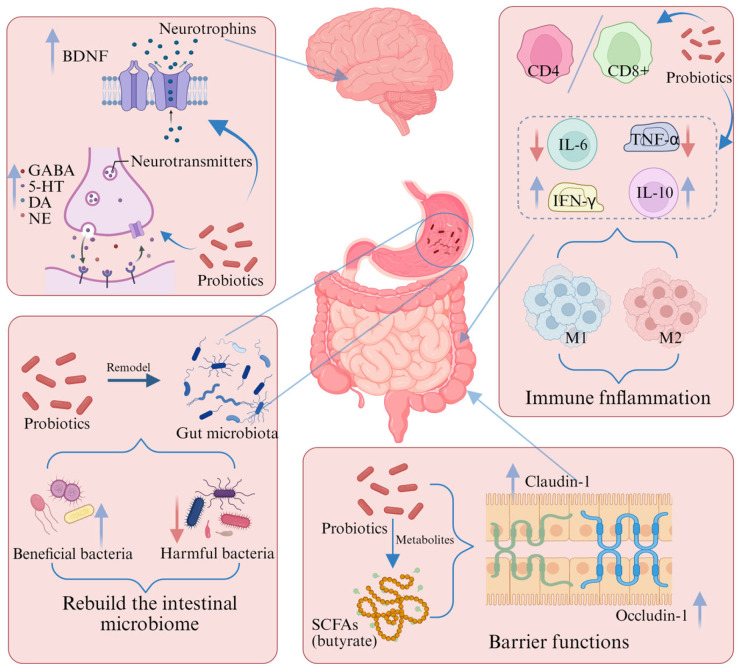
Schematic Diagram of Potential Mechanisms for Probiotic Treatment of Parkinson’s Disease. By regulating the balance of gut microbiota, it produces short-chain fatty acids (such as butyrate) to enhance intestinal barrier function. Simultaneously, it modulates immune inflammatory responses (promoting M2 macrophages and the anti-inflammatory factor IL-10), increases neurotrophic factors (such as BDNF) and neurotransmitters, thereby exerting neuroprotective effects. (Curved blue arrows and dark blue arrows represent different mechanisms of action for probiotics. Short straight light blue arrows indicate promotion, while short straight red arrows indicate inhibition. Long straight blue arrows pointing to organs denote different mechanisms acting on different sites). The figure was created in Biorender (Wang, L. 2026 https://BioRender.com/hal7ub7).

The pathogenesis of PD is closely linked to dysregulation of the gut microbiota-brain axis (MGBA). When the MGBA is disrupted, gut microbiota imbalance occurs, immune inflammatory responses become abnormally activated, and neural signaling pathways are disrupted. These changes intertwine and collectively drive the pathological progression of PD. In recent years, probiotics have emerged as a highly promising therapeutic approach for Parkinson’s Disease (Table 3). Consuming specific probiotics can reshape the gut microbiota structure, increase beneficial bacteria, suppress harmful bacteria growth, and restore intestinal microecological homeostasis. They also modulate immune responses, reduce neurotoxic effects of inflammatory factors, optimize neurotransmitter synthesis and transmission, and improve neurosignaling along the gut-brain axis (Figure 2). This series of positive changes holds promise for PD patients, offering new therapeutic hope to effectively alleviate symptoms and potentially slow disease progression.

### 3.1. Microbial Ecological Remodeling

The starting point of probiotic intervention lies in reshaping the disrupted gut microbiota structure in Parkinson’s Disease. Studies have shown that specific probiotic strains can effectively inhibit the growth of pathogenic bacteria such as *Desulfovibrio* (a genus that produces pro-inflammatory hydrogen sulfide) through mechanisms like competitive colonization and the secretion of antimicrobial peptides. Concurrently, they significantly increase the abundance of beneficial bacteria like *Akkermansia*, *Bifidobacterium*, and *Lactobacillus* [123]. *Akkermansia* is closely associated with improvements in intestinal barrier function and immunomodulation. This suggests that the benefits of probiotics may be partially achieved by modulating the host’s native beneficial microbiota, highlighting their systemic impact on the microecosystem [124]. This ecological restructuring not only restores microbial diversity but, more critically, optimizes its overall metabolic functionality. Among these, short-chain fatty acids represent the most pivotal metabolic products. Strains such as *Bifidobacterium* breve and *Clostridium butyricum* are highly efficient at fermenting dietary fiber, leading to substantial production of butyrate, acetate, and propionate [125]. At the mechanistic level, researchers have discovered that certain specific probiotic strains (e.g., *Lactococcus lactis*) can secrete a small molecular peptide that specifically inhibits the fibrillation process of α-synuclein, thereby preventing the formation of toxic aggregates [125].

### 3.2. The Driving Role of Probiotic Metabolites

As mentioned earlier, probiotic fermentation of dietary fiber produces short-chain fatty acids, among which acetate, propionate, and butyrate serve as the most crucial signaling molecules mediating the “gut-brain dialogue”. They not only serve as an energy source for intestinal epithelial cells but also enter the circulatory system, exerting systemic and central nervous system protective effects through multiple mechanisms [126]. Recent studies have demonstrated that probiotic intervention can significantly increase the concentrations of butyrate, acetate, and propionate in PD mice [127]. Simultaneously, butyric acid supplementation significantly mitigates damage to dopaminergic neuronal cell lines induced by lipopolysaccharide and TNF-α. This mechanism has been demonstrated to involve both the inhibition of histone deacetylase activity and the activation of the Nrf2 antioxidant pathway—the latter being a key defense system for cellular resistance against oxidative stress [128]. Direct injection of sodium propionate into the lateral ventricles of PD model rats significantly reduced glial cell activation and astrocytic proliferation in the substantia nigra region while lowering levels of proinflammatory factors TNF-α and IL-6. This effect was partially mediated through activation of the G protein-coupled receptor GPR43 on microglia, demonstrating that SCFAs can directly act on the brain to exert anti-neuroinflammatory effects [129].

### 3.3. Reinforcing Physical Barriers

Probiotics and metabolites can jointly function through various mechanisms, significantly strengthen the advocacy of physical barriers, and indirectly benefit the integrity of the blood-brain barrier, thus effectively preventing peripheral pathological factors from entering the systemic circulation and central nervous system. *Lactobacillus rhamnosus* LGG treatment can significantly up-regulate the mRNA and protein expression levels of key tight junction proteins Occludin and ZO-1 in colon tissue of PD mice, and at the same time reduce serum levels of endotoxin and inflammatory markers, effectively reducing intestinal permeability [130]. Metabolites of probiotics activate GPR109A receptors in intestinal epithelial cells, which in turn up-regulate the expression of closure proteins [131]. At the blood-brain barrier level, PD model rats treated with *Lactobacillus plantarum* PS128 have significantly improved the leakage of the blood-brain barrier in the substantia nigra region of the brain, which may indirectly stabilize the function of the blood-brain barrier by reducing systemic inflammation and reducing the damage of pro-inflammatory cytokines to the endothelial cells of the blood-brain barrier [132]. At the same time, probiotics can regulate the expression of vascular endothelial growth factor in brain through vagus nerve pathway, thus enhancing the integrity of blood-brain barrier [133]. In addition, long-term supplementation of probiotics can significantly increase the expression of tight junction protein in brain microvascular endothelial cells and maintain the homeostasis of blood-brain barrier by regulating the activity of microglia [134].

### 3.4. Regulation of Neurotrophic Factors and Neurotransmitters

The ultimate protective effect of probiotics manifests in their direct or indirect regulation of central nervous system function, which represents the final link in their therapeutic action against Parkinson’s Disease. Its primary functions include promoting the expression of neurotrophic factors, regulating neurotransmitter balance [135], and even directly influencing neuronal survival. These effects are partially mediated through the MGBA. Concurrently, the expression of brain-derived neurotrophic factor (BDNF) and glial cell-derived neurotrophic factor (GDNF) is upregulated [136]. *Bifidobacterium breve* CCFM1067 promotes GDNF expression in the substantia nigra region of mouse brains by increasing gut-derived serotonin (5-HT) and subsequently activating vagus nerve-dependent pathways, ultimately protecting dopaminergic neurons from damage [137]. *Lactobacillus rhamnosus* increases the utilization of tryptophan, the precursor of 5-HT, in the colonic mucosa and brain of mice, and elevates central 5-HT levels [138]. Beyond monoaminergic neurotransmitters, probiotics can also modulate other neurotransmitter systems. Researchers found that supplementing PD model mice with a composite probiotic containing *Lactobacillus* and *Bifidobacterium* reversed disease-induced glutamate/GABA imbalance in the cortex and hippocampus, reducing elevated glutamate levels. This may help mitigate excitotoxicity and protect neurons [132].

## 4. Effects of Medicinal Plant Polysaccharides on Parkinson’s Disease

**Figure 3:**
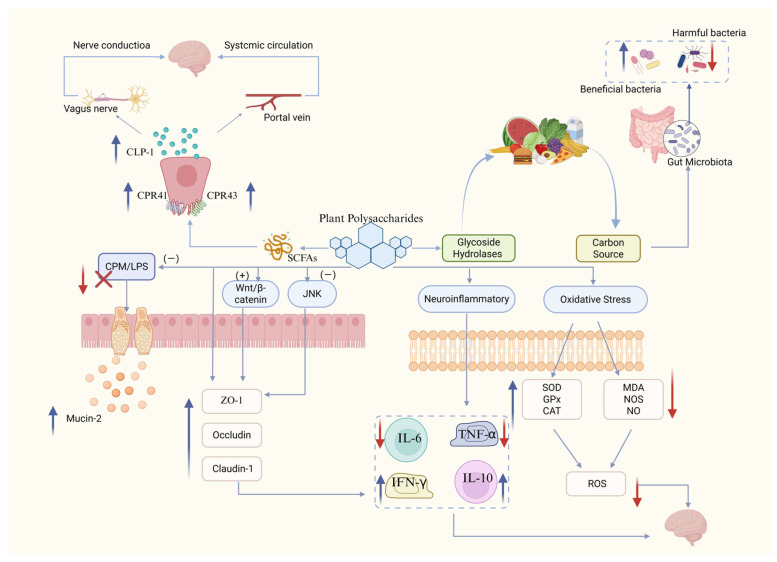
Schematic Diagram of Potential Mechanisms for Plant Polysaccharides in Treating Parkinson’s Disease. The core pathway begins in the gut: plant polysaccharides serve as a carbon source, metabolized by gut microbiota (via glycoside hydrolases) to produce short-chain fatty acids (SCFAs). SCFAs exert effects both locally in the gut—by activating GPR41/43 receptors in intestinal epithelial cells and promoting glucagon-like peptide-1 (GLP-1) release, with signals transmitted to the brain via the vagus nerve—and systemically, as active metabolites like SCFAs enter systemic circulation through the portal vein. At the intestinal level, polysaccharides and SCFAs can “reshape the gut microbiota” by suppressing harmful bacteria and promoting beneficial bacteria, while also “enhancing intestinal barrier function” through upregulating the expression of tight junction proteins (ZO-1, Occludin, Claudin-1). At the systemic and cerebral levels, their primary effects manifest in “modulating immune and inflammatory responses” (downregulating pro-inflammatory factors IL-6, TNF-α, IFN-γ, and potentially upregulating anti-inflammatory factor IL-10) and “alleviating oxidative stress” (enhancing antioxidant enzyme activity of SOD, GSH-Px, CAT, and reducing oxidative damage markers MDA, NOS/NO). Furthermore, the diagram suggests potential effects on neural function through pathways such as Wnt/β-catenin regulation. (Blue arrows indicate promotion/positive effects; red arrows indicate inhibition/negative effects; red ‘X’s represent blocking/inhibition of specific processes). The figure was created in BioRender (Wang, L. (2026) https://BioRender.com/hn566he).

In dietary intervention strategies for regulating the MGBA, prebiotics are equally important as probiotics. Prebiotics are defined as dietary components that are not digested by the host but selectively promote the growth or activity of one or more beneficial bacteria in the gut, thereby conferring health benefits to the host. Compared to traditional prebiotics such as inulin and fructooligosaccharides, polysaccharides derived from medicinal plants—which share a dual status as both food and medicine—exhibit unique advantages. Not only do they serve as “exclusive nutrient sources” that promote the growth of beneficial bacteria, but their complex structures and diverse bioactivities also enable them to directly participate in regulating host immunity, metabolism, and barrier functions, acting as “global regulators”. The biological activity of plant polysaccharides stems from their remarkable structural diversity (Table 4). Unlike conventional prebiotics with relatively simple structures, they are high-molecular-weight polymers composed of multiple monosaccharides (such as glucose, galactose, arabinose, rhamnose, and mannose) linked by various types of glycosidic bonds (e.g., α- or β-1,3/1,4/1,6 bonds), often featuring branched structures. This vast variation in molecular weight, monosaccharide composition, glycosidic bonds, and spatial conformation determines their functional specificity and broad range of target sites (Figure 3).

### 4.1. Enhancing Microbial Community Diversity

Alpha diversity of the gut microbiota serves as the gold standard indicator for assessing microbial health and stability. Plant polysaccharides exert profound effects on the composition and organization of the gut microbiota. They can serve as carbon sources, enrich beneficial gut microorganisms, or regulate the development, activity, and synthesis of microbial metabolites. Additionally, in a rat model of type 2 diabetes, intervention with *Phellinus linteus* polysaccharides increased short-chain fatty acid (SCFA) biosynthesis and bile acid (BA) metabolism, thereby promoting GLP-1 secretion. This subsequently enhanced insulin release and reduced blood glucose levels [163]. High-throughput sequencing analysis reveals that *Ziziphus jujuba* polysaccharides can prevent colon cancer by improving cancer-induced dysbiosis in the gut microbiota, demonstrating significant prebiotic effects [164]. The mechanism lies in the fact that *Astragalus* polysaccharides are rich in polysaccharide chains such as (1→4)-glucose and (1→6)-galactose, whereas *Ganoderma lucidum* polysaccharides primarily consist of β-(1→3)/(1→6)-glucan as their main chain. The diversity of this structure necessitates the synergistic utilization of multiple microorganisms with distinct enzymatic profiles to convert polysaccharides into prebiotics beneficial to the host. Metatranscriptomic analysis revealed that following intervention with *Astragalus* polysaccharides and *Codonopsis* polysaccharides, the expression of carbohydrate-active enzyme genes involved in polysaccharide degradation was significantly upregulated in the gut microbiota of mice. This was particularly evident in the GH family from the *Bacteroidetes* phylum and glycoside hydrolases from the *Firmicutes* phylum [165]. During the degradation of kelp polysaccharides, microbial communities first target the readily degradable alginic acid before processing the structurally more complex fucoidans [166]. This synergistic activation of the multi-enzyme system enables microorganisms from different metabolic lineages—such as *Bacteroides*, *Prevotella*, and *Bifidobacterium*—to obtain the carbon sources necessary for their growth. Treatment with donkey-hide gelatin polysaccharides in type 2 diabetic mice significantly reversed alterations in bacterial species and metabolites observed in diseased mice. Furthermore, 16S rDNA sequencing analysis demonstrated that *Angelica sinensis* polysaccharides can reshape gut microbiota composition, thereby reducing joint swelling in rheumatoid arthritis and significantly suppressing anti-CII antibodies and proinflammatory factors [167].

### 4.2. Optimizing Microbial Community Composition

Building upon the enhancement of gut microbiota diversity, plant polysaccharides can further finely regulate the structure of microbial communities at the phylum and genus levels [168]. On one hand, they can specifically enrich beneficial microbial communities. For instance, at the phylum level, plant polysaccharides effectively regulate key microbial ratios. Studies indicate that intervention with *Astragalus* polysaccharides significantly increased the Firmicutes/Bacteroidetes ratio in the intestines of normal mice while markedly reducing the relative abundance of the Proteobacteria phylum [169]. Similarly, in models of gut microbiota dysbiosis and intestinal barrier dysfunction induced by broad-spectrum antibiotics, *bamboo fungus* polysaccharides can restore disrupted gut microbial composition (including regulating the Firmicutes/Bacteroidetes ratio and reducing the relative abundance of harmful phyla such as Proteobacteria, *Enterococcus*, and *Bacteroides*) [170]. *Ganoderma* polysaccharides enhanced the microbial diversity and proportion of *Pseudomonas* and *Bacillus* species, significantly enriching *Ovalibacillus* and *Bacillus uniformis* [171]. Notably, the regulatory effects of different herbal polysaccharides exhibit specificity. Researchers compared the actions of four common herbal polysaccharides and found that *Dioscorea* polysaccharide uniquely increased the relative abundance of *Proteobacteria* while reducing the proportions of *Ascomycota* and Bacteroidetes. This may be related to its distinctive monosaccharide composition [172]. At the species level, the enrichment effect of Chinese herbal polysaccharides on specific beneficial bacteria is more pronounced. Multiple studies have confirmed their promotion of *Akkermansia:* research reports indicate that ginger polysaccharides significantly increase the relative abundance of *Akkermansia* in the intestines of obese mice [173]. Following intervention with prickly pear polysaccharides, the abundance of *Akkermansia* increased, accompanied by a marked improvement in the thickness of the intestinal mucus layer [174]. Moreover, the regulatory effect of Chinese herbal polysaccharides on short-chain fatty acid-producing bacterial communities is particularly pronounced. Following intervention with *Astragalus* polysaccharides, the abundance of short-chain fatty acid-producing bacterial communities—such as the genera *Clostridium*, *Faecalibacterium*, *Akkermansia*, *Lactobacillus*, and *Ruminococcus*—all exhibited an upward trend. Fecal analysis revealed that following supplementation with *Astragalus* polysaccharides, acetic acid, propionic acid, and butyric acid levels significantly increased in mice with adenine-induced chronic kidney disease (CKD) models (Ade) [175]. Similarly, it was found that *Ganoderma lucidum* polysaccharide intervention increased the abundance of *Lactobacillus*, *Bacteroides*, and *Bacteroides fragilis*, while also elevating levels of short-chain fatty acids (SCFAs) in feces [176]. While promoting the growth of beneficial bacteria, plant polysaccharides effectively suppress potential pathogens through multiple mechanisms. *Astragalus* polysaccharides can significantly reduce the abundance of *Shigella*, a potentially pathogenic intestinal bacterium. This effect is closely associated with their promotion of *Lactobacillus* growth and lactic acid secretion [177]. *Rhubarb* polysaccharide supplementation modulates the gut microbiota by enriching beneficial genera such as *Lactobacillus* and *Akkermansia*, while reducing pathogenic genera like *Clostridium difficile* and *Desulfovibrio* [178].

### 4.3. Enhancing the Physical Barrier Function of the Intestinal Tract

An intact intestinal barrier serves as a physical defense against the translocation of intestinal endotoxins, such as lipopolysaccharide (LPS), which can trigger systemic neuroinflammation. Plant polysaccharides reinforce this defense through multiple pathways. Firstly, during their degradation by gut microbiota, carbohydrate-active enzymes and genes associated with butyrate production are upregulated. The resulting butyrate serves as a crucial energy source for colonic epithelial cells, which significantly enhances the mRNA expression of tight junction proteins (e.g., ZO-1, Occludin, Claudin-1) and reduces inflammatory responses, thereby improving intestinal barrier function [179,180]. Secondly, certain polysaccharides mitigate cyclophosphamide (CPM)-induced intestinal mucosal damage and increase the number of jejunal goblet cells, thereby promoting the synthesis and secretion of mucin-2 and thickening the protective mucus layer [181]. The synergistic action of both components effectively reduces intestinal permeability and repairs the “leaky gut.” Plant polysaccharides exhibit significant specificity in regulating intestinal tight junction proteins. Studies indicate that *Atractylodes* polysaccharides effectively enhance immune organ indices and blood cell counts, stimulate cytokine secretion, and promote the expression of occludin and tight junction protein-1 (ZO-1), while significantly reducing lipopolysaccharide-induced epithelial permeability [160]. Mushroom polysaccharides rich in β-glucan significantly upregulate the gene expression of tight junction proteins in lipopolysaccharide (LPS)-stimulated Caco-2 cells, demonstrating their potential to improve intestinal barrier dysfunction [182]. Additionally, longan pulp polysaccharides can prevent intestinal mucosal damage by increasing the expression of mucin 2, tight junction protein ZO-1, claudin-1, claudin-4, and adhesion junction protein E-cadherin [183]. It is noteworthy that the mechanisms of action vary among different plant polysaccharides. Comparative studies reveal that *Codonopsis* polysaccharides promote epithelial cell proliferation and differentiation by bidirectionally regulating the Wnt/β-catenin signaling pathway, thereby upregulating the transcription and expression of tight junction proteins such as ZO-1 and Occludin. *Astragalus* polysaccharides may mitigate the degradation of tight junction proteins by inhibiting JNK phosphorylation [184]. *Ganoderma lucidum* polysaccharides can also maintain intestinal barrier function by increasing the expression of Wnt/β-Catenin and Lrp5 proteins [185]. *Lycium* polysaccharides significantly prolonged the survival time of septic mice by regulating inflammatory responses and inhibiting JNK pathway activation, while also improving the animals’ digestive function and intestinal barrier integrity [186]. Regarding the mucosal barrier, plant polysaccharides demonstrate significant promotional effects. Research indicates that *Hericium erinaceus* polysaccharides can enhance levels of secretory immunoglobulin A (SIgA) and cytokines secreted by the intestinal mucosal immune system in Muscovy ducks, while simultaneously repairing damaged intestinal mucosal immune barriers [187]. Further research confirms that *Artemisia annua L*. polysaccharides can enhance the intestinal physical barrier by downregulating serum ET levels and increasing the villus height/crypt depth (VH/CD) ratio and ZO-1 mRNA levels in broiler chickens challenged with *Escherichia coli*. Additionally, they elevate VH/CD ratios and mRNA levels of Occludin, ZO-1, and Mucin-2 in non-challenged broilers [188]. Experimental evidence also confirms that plant polysaccharides indirectly enhance barrier function by regulating microbiota metabolism. Metabolomics analysis revealed that raspberry polysaccharide intervention increased butyrate concentrations in mouse intestines, showing a significant positive correlation with enhanced tight junction protein expression [189]. The anti-inflammatory effects of plant polysaccharides play a crucial role in barrier protection. Studies have found that polysaccharides derived from rapeseed and algae can significantly reduce serum levels of endotoxin, DAO, and D-lactic acid [190,191]. Particularly noteworthy is the multi-target synergistic mechanism of Chinese herbal polysaccharides. Comprehensive studies indicate that polysaccharides not only directly upregulate tight junction protein expression but also promote short-chain fatty acid (SCFA) production by modulating the gut microbiota while simultaneously inhibiting the NF-κB inflammatory pathway, thereby establishing a triple protective mechanism [191]. Recent research further reveals that *Astragalus* polysaccharides can enhance intestinal barrier function systemically by modulating the gut microbiota-bile acid-FXR axis [192].

### 4.4. System Regulation of Host Immunity and Metabolism

The systemic regulatory role of plant polysaccharides in host immunity and metabolism demonstrates their multifaceted value as “immunometabolic modulators”. This regulation is achieved not only through indirect pathways mediated by the gut microbiota but also through the direct effects of polysaccharide molecules on immune cells. Multiple studies have revealed the direct immunomodulatory mechanisms of plant polysaccharides at the molecular level. For instance, *Astragalus* polysaccharides can dose-dependently inhibit LPS-induced NF-κB p65 nuclear translocation in RAW264.7 macrophages, thereby reducing the secretion of TNF-α and IL-6 [193], and can also reduce IL-1β maturation and release by inhibiting NLRP3 inflammasome assembly and decreasing caspase-1 activity [194]. Research has also reported that *Astragalus* polysaccharides can reduce chondrocyte apoptosis by activating the thioredoxin system and inhibiting the ASK1/p38 signaling pathway [195]. Additionally, in MPTP-induced silkworm Parkinson’s Disease models, administration of *Lycium* polysaccharide improved motor function, dopamine levels, and TH activity, while simultaneously reducing oxidative damage [196]. The exocarp polysaccharides from red tangerines significantly alleviated Parkinson’s Disease symptoms and improved behavioral deficits by modulating the HDAC6/NLRP3 pathway in Parkinson’s Disease mice [197]. *Yuzhu* polysaccharides significantly enhanced the activity of antioxidant enzymes superoxide dismutase (SOD), glutathione peroxidase (GSH-Px), and catalase (CAT), reduced malondialdehyde (MDA) levels, and reversed the expression of oxidative stress-related proteins Nrf2 and Keap1 in the substantia nigra pars compacta (SNc) of PD mice. Furthermore, *Yuzhu* polysaccharide treatment significantly suppressed MPTP-induced neurotoxicity, markedly alleviated weight loss and motor dysfunction, and mitigated the loss of dopaminergic neurons in the SNc of PD mice. Concurrently, it reduced the expression levels of pro-apoptotic proteins Bax and cleaved caspase-3 while elevating the expression of the anti-apoptotic protein Bcl-2 [198]. The effects of plant polysaccharides on immune cell differentiation have been validated in multiple studies. Treatment with *Astragalus* polysaccharides significantly improved immune cell differentiation in cyclophosphamide-induced immunosuppressed mice, restoring the numbers of CD4+ T cells, CD8+ T cells, CD19+ B cells, and F4/80 + CD11B+ macrophages to normal levels [199]. At the metabolic level, *Astragalus* polysaccharides significantly improve polyunsaturated fatty acid metabolism, balancing the ratio of linoleic acid to α-linolenic acid in serum. Additionally, different plant polysaccharides exhibit unique immunomodulatory properties. Research indicates that *Ganoderma lucidum* polysaccharide peptides significantly elevate serum levels of TNF-α, IFN-γ, IL-2, and immunoglobulin A in immunosuppressed mice [200]. *Lycium* polysaccharides exert anti-inflammatory effects by regulating macrophage polarization toward the M2 type through inhibiting STAT1 phosphorylation (65.3% reduction) and promoting STAT6 phosphorylation (2.2-fold increase) [201]. The mechanism by which plant polysaccharides influence systemic immunity through modulating gut microbiota has also been extensively studied. Research indicates that *Poria cocos* polysaccharides can restore intestinal and pulmonary microbial diversity in cyclosporine A-induced immunosuppressed mice, significantly improving lung tissue damage. This effect is closely associated with the normalization of serum metabolite profiles [202]. Recent studies indicate that yam polysaccharides significantly enhance the efficacy of PD-1 monoclonal antibodies in treating colorectal cancer by inhibiting M2 macrophage polarization through reducing the abundance of the gut microbial metabolite deoxyribose [203]. *Panax ginseng* polysaccharides can stimulate immune cells and promote the release of immune factors, thereby regulating immune function. GPNE-I, a component of *Panax ginseng* polysaccharides, promotes lymphocyte proliferation and acts as an immunomodulator [151]. Advances in multi-omics research have provided more comprehensive evidence for the systemic regulatory effects of plant polysaccharides. By integrating metagenomics, metabolomics, and transcriptomics data, a holistic network was constructed showing how mulberry leaf polysaccharides regulate the “gut microbiota-metabolite-immune axis”. This revealed that they can influence hepatic immune responses by promoting the growth of butyrate-producing bacteria and modulating bile acid metabolism [204]. Similarly, the dynamic changes in gut microbiota mediated by shiitake mushroom polysaccharides effectively regulate lipid metabolism in high-fat diet (HFD)-fed obese mouse models [205].

## 5. Synergistic Effects of Plant Polysaccharides and Probiotics

Plant polysaccharides, as high-quality prebiotics, significantly enhance the survival capacity, colonization efficiency, and metabolic activity of probiotics in the complex gastrointestinal environment through multiple mechanisms. This “empowering” effect forms the basis for the synergistic action of synbiotics.

### 5.1. Synbiotics Enhance the Colonization and Vitality of Intestinal Probiotics

Multiple in vivo studies confirm that the combination of Chinese herbal polysaccharides and probiotics effectively enhances the survival and colonization levels of the latter in disease model intestines, directly contributing to the restoration of the intestinal barrier. The co-administration of *Lactobacillus plantarum* and *Astragalus* polysaccharides significantly improves intestinal health in mice with antibiotic-associated diarrhea (AAD). This study not only observed significantly higher levels of lactobacilli in the intestines of the synbiotic group compared to the single probiotic group, but more importantly, immunofluorescence and Western blot analyses revealed that synbiotic treatment significantly upregulated the expression of tight junction proteins (such as Occludin and ZO-1) in colonic tissue. Mechanistic studies suggest this barrier repair effect may be partially mediated through activation of the TGF-β/Smad signaling pathway. Spearman correlation analysis further revealed that increased gut lactobacilli abundance positively correlated with barrier function indicators while negatively correlating with permeability factors like serum endotoxin levels, directly demonstrating the link between enhanced probiotic colonization and improved host physiology [206]. A combination of *Lycium* polysaccharides and Japanese kelp polysaccharides was obtained via enzyme-assisted acid extraction, yielding two pectin-type *Lycium* polysaccharides rich in rhamnogalacturonic acid I and two fucoidan-type Japanese kelp polysaccharides. These were paired to form four proportional mixtures. This approach efficiently targets and promotes the proliferation of *Bifidobacteria*, *Lactobacillus*, and *Bacteroides*, enhancing the production of short-chain fatty acids and non-short-chain fatty acid health-related metabolites. It also stimulates the accumulation of butyrate-producing bacteria and their metabolites [207]. A study on the synergistic effects of alginate polysaccharides and *Lactobacillus plantarum* in colitis-affected mice revealed that compared to monotherapy with probiotics, the combined intervention significantly increased the abundance of beneficial bacteria such as *Lactobacillus* and *Bifidobacterium* in the gut of colitis mice while more effectively suppressing the proliferation of pathogenic bacteria. This reshaping of the microbial community structure occurred concurrently with reduced colonic inflammation scores and lessened histological damage, demonstrating the synbiotic’s ability to enhance probiotic efficacy in disease conditions by promoting the colonization of beneficial bacteria in DSS-induced colitis [208]. Probiotic powder containing *Poria cocos* polysaccharides demonstrated superior efficacy compared to probiotic powder without *Poria cocos* polysaccharides in improving immune regulation and gut microbiota in mice with antibiotic-associated diarrhea. It significantly increased Proteobacteria such as *Sutterella* and reduced Bacteroidetes such as Muribaculaceae, effectively suppressing dysbiosis induced by diarrhea in mice [208]. Feeding a diet containing both probiotics and a *Astragalus* polysaccharides resulted in significant differences in *Lactobacillus*, *Bacillus cereus*, and *Escherichia coli* counts compared to diets containing either probiotics or *Astragalus* polysaccharides alone. The numbers of *Lactobacillus* and *Bifidobacterium* increased, while *Escherichia coli* counts decreased [209]. Fermentation of bamboo shoot polysaccharides with *Streptococcus thermophilus* produced fermented milk that significantly promoted the proliferation of *Streptococcus thermophilus*, *Lactobacillus* bulgaricus, and *Bifidobacterium* longum F2, while enhancing acetic acid production and imparting a distinctive flavor [210].

### 5.2. Synbiotics Optimize Microbial Metabolic Output

Metabolites produced by gut microbiota serve as key mediators regulating host physiology, with short-chain fatty acids (SCFAs) being one of the most important “messengers” among them. The synergistic effect of plant polysaccharides and probiotics lies in significantly enhancing the “production” of SCFAs and optimizing their “composition spectrum,” which forms the metabolic basis for their systemic probiotic effects. When probiotics are ingested alone, their ability to produce SCFAs is constrained by available substrates. Conversely, when polysaccharides are ingested alone, the depth of fermentation and the spectrum of products are highly dependent on the host’s inherent microbial structure—a structure that is often impaired in Parkinson’s Disease. The synbiotics strategy ingeniously combines the strengths of both approaches. Research reveals that *Lactobacillus plantarum* utilizes its abundant glycosidase enzymes (such as α-galactosidase and β-mannanase) to preliminarily degrade the complex side chains within *Astragalus* polysaccharides. These degradation products not only serve as nutrients for the bacteria themselves but, more importantly, provide more readily available substrates for specialized SCFA-producing commensal bacteria in the gut, such as *Propionibacterium* and Rectobacterium [211,212,213]. In vitro co-culture and colitis mouse models demonstrated that compared to supplementation with *Astragalus* polysaccharides alone, synbiotic intervention increased concentrations of total SCFAs, butyrate, and propionate in colonic contents while simultaneously upregulating the abundance of key metabolic genes such as butyrate kinase (buk) within the microbiota. In rat models, the combination of Crataegus microphylla crude polysaccharides with probiotics (containing *Bifidobacterium* and *Lactobacillus*) significantly outperformed single components in increasing total intestinal acetate, propionate, and butyrate levels. Metagenomic analysis revealed that the synbiotic regimen significantly upregulated gene clusters associated with acetyl-CoA transferase and butyrate kinase in the gut microbiota. These changes were strongly correlated with elevated SCFA levels and reduced serum inflammatory markers, directly linking synergistic metabolism to host benefits at the functional gene level [214]. During fermentation using polysaccharides, probiotics themselves produce organic acids (such as lactic acid), lowering the local pH. This further shapes a microenvironment conducive to the growth of SCFA-producing bacteria (many of which are strict anaerobes) and may inhibit pathogenic bacteria. Research indicates that Sanghuang Feng polysaccharides effectively promote the proliferation of *Lactobacillus plantarum* and *Lactobacillus rhamnosus* while lowering the culture medium pH, which suggests that these probiotics utilize the polysaccharides as a carbon source for fermentation and SCFA production [215]. The synergistic interaction between beneficial gut microbiota and shiitake mushroom polysaccharides generates substantial amounts of short-chain fatty acids (SCFAs), particularly butyrate, propionate, and acetate. Among these, butyrate serves as the primary energy source for colonic epithelial cells and is crucial for maintaining intestinal barrier function [216].

### 5.3. Synbiotics Protect the Intestinal Barrier

“Leaky gut” and “blood-brain barrier leakage” are two key interconnected pathways in PD pathology. The synergistic effects of medicinal plant polysaccharides and probiotics provide “dual reinforcement” for these biological barriers. When traditionally extracted polysaccharides undergo fermentation by specific probiotics, their structure and biological activity may undergo beneficial transformations, thereby enhancing their capacity to protect the intestinal barrier. The study demonstrated that *Acacia* seed polysaccharides fermented by *Bacillus thuringiensis* exhibited enhanced protective effects on the intestinal epithelium, improved barrier repair, and extended the lifespan in a Drosophila intestinal inflammation model. This research indicates that probiotic fermentation not only degrades polysaccharides to enhance their bioavailability but may also modify their higher-order structures (e.g., preserving or altering triple-helix conformations), thereby strengthening their interactions with intestinal epithelial cells or immune receptors. This process yields enhanced barrier repair and systemic health benefits [217]. The fermented product of shiitake mushroom polysaccharides by probiotics significantly reverses the LPS-induced downregulation of tight junction protein expression. For example, compared with the LPS model group, the fermented product-treated group showed increased mRNA expression of ZO-1, Occludin, and Claudin-1 [216]. Damage to the intestinal barrier is often accompanied by abnormal infiltration and activation of immune cells. Synbiotics can mitigate inflammation’s assault on the barrier at its source by synergistically regulating immune cell function. Research indicates that treatment with probiotics and *Polygonatum* polysaccharides significantly suppresses inflammation-induced migration in RAW 264.7 macrophages under high-fat conditions. This demonstrates that fermented polysaccharides effectively reduce the attraction of inflammatory signals, thereby decreasing immune cell infiltration into intestinal tissues at the source and mitigating local inflammatory stress on epithelial cells [218]. The integrity of the intestinal barrier relies on the mucus layer above the epithelium, and synbiotics synergistically promotes goblet cell regeneration and mucus secretion. Fucoidan and *Lactobacillus plantarum* most effectively stimulate goblet cell regeneration in zebrafish colitis, thereby aiding in the restoration of the mucus layer that protects the intestinal epithelium. Additionally, synbiotics significantly improved crypt structural disruption and submucosal edema induced by DSS [218]. Therefore, the improvement in intestinal barrier function directly reduces the entry of pathogen-associated molecular patterns (such as lipopolysaccharide LPS) and inflammatory mediators of intestinal origin into the circulatory system.

### 5.4. Synbiotics Restores Immune Homeostasis

The gut is the largest immune organ in the human body, and the onset and progression of PD are closely associated with immune dysregulation in both the gut and systemic immune systems. Medicinal plant polysaccharides and probiotics jointly act on the innate and adaptive immune systems, synergistically reshaping immune homeostasis. The establishment of immune tolerance begins with the proper development of immune organs and the phenotypic regulation of antigen-presenting cells. The effects of *Astragalus* polysaccharides and a composite probiotic (containing *Lactobacillus* and *Bacillus cereus*) on the immune systems of chicks were investigated. The study revealed that compared to the group treated with *Astragalus* polysaccharides alone, the relative weights of the bursa of Fabricius and spleen in the synbiotic group increased significantly. More importantly, the synbiotic treatment synergistically promoted the induction of regulatory T cells (Tregs). This synergistic enhancement of both central and peripheral immune organs laid the foundation for establishing a stable immune tolerance environment [209]. At a finer molecular level, probiotics were encapsulated within durian peel polysaccharide gel and administered to zebrafish. Results demonstrated that this synbiotic diet synergistically and significantly upregulated key immune genes in zebrafish, including pro-inflammatory factor interleukin-1β (IL1B), antimicrobial peptide lysozyme (LYZ), tumor necrosis factor-alpha (TNF-α), and antioxidant enzyme superoxide dismutase (SOD). This broad spectrum of immune gene activation indicates that synbiotics do not simply suppress or activate immunity. Instead, they enhance the body’s immune surveillance and clearance capabilities in a coordinated manner while preventing excessive inflammatory damage by elevating antioxidant levels [219]. Given the widespread imbalance in pro-inflammatory cytokine levels in PD patients, synbiotics can synergistically regulate this critical network. Research indicates that co-administration of *Crataegus pinnatifida* crude polysaccharides with specific probiotics not only modulates the gut microbiota in Wistar rats but also synergistically influences host tryptophan metabolism. This approach elevates levels of immunomodulatory indole derivatives and optimizes serum cytokine profiles. Such coordinated regulation of immunometabolites serves as a vital bridge linking microbiome alterations to host immune phenotypes [214]. In studies on broiler chickens infected with Escherichia coli, the combined application of *Astragalus* polysaccharides and probiotics (*Bacillus subtilis* and *Lactobacillus*) was more effective than single-component treatments in reducing diarrhea rates and mortality, as well as alleviating intestinal pathological damage. Mechanistically, the synbiotic significantly increased mRNA expression of the anti-inflammatory cytokine IL-10 in broiler intestines while markedly suppressing TLR4 mRNA expression. Concurrently, expression of the pro-inflammatory cytokine IL-2 was moderately upregulated. This pattern indicates that the synbiotic treatment synergistically “shuts down” excessive pro-inflammatory signaling pathways (TLR4), “activates” protective anti-inflammatory signaling (IL-10), and “maintains” essential immune responses (IL-2), thereby achieving precise immune balance [220]. The combination of probiotic *Lactobacillus plantarum* S58 and prebiotic barley β-glucan demonstrated significant synergistic neuroprotective effects in a mouse model of colitis. Synbiotic therapy not only more effectively suppressed pro-inflammatory cytokines in the colon and serum, alleviating intestinal inflammation, but also synergistically blocked excessive activation of hippocampal microglia. This slowed synaptic protein degradation and neuronal apoptosis, thereby improving cognitive deficits in the mice [221]. Compared to using probiotics or *Achyranthes* polysaccharides alone, their combined use enhances positive effects more than individual supplements. Not only are serum immunoglobulin A (IgA) and interleukin-2 (IL-2) concentrations higher, but jejunal villus height (VH) is also improve [222]. In summary, the synbiotic combination of plant-derived medicinal polysaccharides and probiotics establishes a comprehensive, closed-loop functional network. This network progresses from “empowering probiotics” to “optimizing metabolic output,” then to “strengthening dual barriers” and “restoring immune homeostasis,” ultimately achieving “integrated neuroprotection” through the gut-brain axis. This multi-targeted, systemic regulatory strategy not only aligns closely with the multifactorial and complex pathological characteristics of PD but also paves a highly promising new pathway for developing PD intervention strategies based on the gut microbiome.

## 6. Potential and Challenges of Synbiotics in Parkinson’s Disease Treatment

### 6.1. Heterogeneity of Gut Microbiota

The composition of the microbiome varies greatly between individuals, influenced by multiple factors including genetic background, age, gender, geography, long-term dietary habits, lifestyle (such as exercise and stress), and medications (particularly antibiotics and PD drugs themselves), forming a highly complex and dynamic ecosystem [223]. This heterogeneity manifests not only in species composition but also in functional genes and metabolic pathways, directly determining the host’s response to dietary interventions. Metagenomic analysis reveals significant individual variation in the gut microbiota of PD patients. Compared to healthy controls, however, they collectively exhibit a reduction in genes involved in short-chain fatty acid (SCFA) synthesis and an increase in metabolic pathways associated with pathogenic bacteria [224]. More importantly, the dysbiosis patterns observed in PD patients are not uniform but can be categorized into distinct “gut types” or “clusters”. These clusters exhibit varying degrees of association with disease severity, progression rate, and even non-motor symptoms such as constipation. For instance, clusters dominated by *Bacteroides* species may correlate with milder motor symptoms, whereas clusters characterized by increased abundance of certain *Ruminococcus* species are associated with more severe disease states [225]. This implies that a standard formula of synbiotics administered to PD patients with different gut types may yield varying outcomes. It was discovered that healthy individuals exhibit a continuum of colonization responses ranging from “permissive” to “complete resistance” to the same commercial probiotic (containing 11 bacterial strains). This resistance is primarily determined by the structure and function of the individual’s baseline microbiota, preventing the probiotic from effectively colonizing or altering the existing gut flora in resistant individuals [226]. The efficacy of probiotics in synbiotics depends on their ability to colonize or exert transient effects within the complex intestinal environment, while the utility of prebiotics (such as medicinal plant polysaccharides) hinges on the presence of bacteria within the host microbiota capable of specifically fermenting and utilizing them. A clinical trial found that supplementation with *Lactobacillus fermentum* improved constipation symptoms in some PD patients, though responses varied. Subsequent analysis suggested that patients with a higher pre-treatment abundance of *Prevotella*, which produces mucin-degrading enzymes, may have experienced an altered intestinal environment that hindered the initial adhesion of certain probiotics [118]. Another study on *Bifidobacterium longum* also observed that its colonization success rate in PD patients was not 100%, with those who successfully colonized showing more significant reductions in inflammatory markers. Medicinal plant polysaccharides possess complex structures, requiring specific enzyme systems for their degradation [119]. For example, the fermentation of *Astragalus* polysaccharides is highly dependent on *Bacteroides* species possessing corresponding polysaccharide degradation genes in the gut. The utilization of *Ganoderma lucidum* polysaccharides may be associated with certain species of the genus *Ruminococcus* and the family Helicobacteraceae. The abundance and diversity of functional microbial communities with polysaccharide degradation potential in the intestines of PD patients are generally reduced and exhibit significant individual variation. Therefore, when administered the same *Ganoderma lucidum* polysaccharides, they may be effectively fermented into anti-inflammatory short-chain fatty acids in Patient A, while in Patient B, they may yield minimal benefits due to the absence of key degrading bacteria, or they may even be converted by other microbial communities into non-beneficial or harmful metabolites. Future approaches should integrate in vitro fermentation models with artificial intelligence prediction to match optimal combinations of medicinal plant polysaccharides and probiotics for PD patients with diverse gut ecological backgrounds.

### 6.2. The Complexity of Medicinal Plant Polysaccharides and the Specificity of Probiotics

The prebiotic activity of medicinal plant polysaccharide is highly dependent on their fine chemical structure, including monosaccharide composition, glycosidic linkage types, molecular weight, branching degree, and spatial conformation [227]. This structural diversity determines the specificity of the enzyme systems required for their degradation, as well as the spectrum of fermentation products generated and their ultimate biological effects [228]. For instance, linear β-(1→3)-glucans (e.g., from *Ganoderma lucidum*) are more readily and rapidly utilized by specific bacterial groups (such as *Bacteroides spp.*), leading to the production of short-chain fatty acids. In contrast, rhamnogalacturonan I-type pectin with complex side chains (e.g., from *Lycium* barbarum) may undergo slower, multi-stage fermentation, sustainably nourishing various acid-producing and mucus-degrading bacteria (such as *Akkermansia* spp. [229]. This specificity in the structure-function-microbiota response relationship poses a core challenge for the precise selection and dosage of plant polysaccharides in synbiotic formulations. Insufficient dosage may fail to effectively activate target metabolic pathways, while excessive dosage could lead to over-fermentation, resulting in bloating, gastrointestinal discomfort, and even adverse effects on the host due to excessive gas production or certain intermediate metabolites (such as D-lactic acid) [230]. Therefore, achieving both safety and efficacy necessitates a personalized and stratified application strategy based on individual characteristics. This approach must fully account for the molecular properties of plant polysaccharides, the metabolic capacity of target probiotics, and the patient’s baseline gut ecology. Probiotics are not a functionally homogeneous group; their health effects are highly strain-specific. Different strains exhibit significant variations in their adhesive and colonization capabilities, the synthesis of metabolites (such as short-chain fatty acids, gamma-aminobutyric acid, and bacteriocins), immunomodulatory properties, and their regulation of host signaling pathways (e.g., Toll-like receptors and G protein-coupled receptors) [231]. For instance, in Parkinson’s Disease models, *Lactobacillus plantarum* PS128 has been shown to increase striatal dopamine levels and improve motor symptoms, while certain strains of *Lactobacillus* or *Bifidobacterium* may focus more on anti-inflammatory effects or barrier repair [232]. Therefore, when constructing synbiotics, the selection of probiotic strains should not be based solely at the genus or species level but should extend to the strain level. Screening should be conducted based on their validated, specific functions relevant to the pathological mechanisms of Parkinson’s Disease (PD), such as inhibiting α-synuclein aggregation, enhancing GDNF expression, and reducing pro-inflammatory cytokines [233]. More critically, the “synergy” pursued in synbiotics is not merely a simple stacking of bacterial species but rather the achievement of “functional complementarity” and “metabolic relay.” An ideal combination should include “primary degraders” (such as certain *Lactobacillus* strains) that can utilize the initial complex structures of plant polysaccharides and provide precursor substances for other bacteria. along with “core acid-producing bacteria” (such as *Clostridium butyricum* and *Faecalibacterium prausnitzii*) that efficiently ferment these precursors to generate substantial amounts of neuroprotective metabolites (e.g., butyrate); as well as “immuno/neuro-regulatory bacteria” capable of directly communicating with the host immune system, strengthening the intestinal barrier, or modulating neurotransmitters. Future research should utilize in vitro co-culture systems, gnotobiotic, and meta-transcriptomics technologies to systematically analyze the metabolic interaction networks between different plant polysaccharides and specific probiotic strains. This approach will enable the design of truly next-generation, metabolically intelligent synbiotics capable of delivering therapeutic efficacy for Parkinson’s disease [234].

## 7. Conclusions

This review systematically demonstrates the potential of “medicinal plant polysaccharide-probiotic synbiotics” as an innovative strategy targeting the microbe-gut-brain axis (MGBA) for Parkinson’s Disease (PD) intervention. The onset and progression of PD are closely associated with MGBA dysregulation, involving multiple interconnected pathways such as gut microbiota disturbance, intestinal barrier disruption (“leaky gut”), pathological α-synuclein propagation, and central nervous system inflammation. Single-agent probiotic or prebiotic interventions face challenges including colonization difficulties and dependence on the host’s existing microbiota, resulting in limited efficacy. Medicinal plant polysaccharides surpass traditional prebiotics due to their structural diversity and multifunctional activities—such as selective probiotic promotion, immunomodulation, antioxidant effects, and direct repair of the intestinal barrier—making them ideal components for constructing highly effective synbiotics. When plant polysaccharides form synbiotics with specific probiotics, they generate significant synergistic effects: polysaccharides provide exclusive nutrition for probiotics, markedly enhancing their survival, colonization, and metabolic activity in the intestinal environment; synergistically promote the yield and composition of beneficial metabolites like short-chain fatty acids, which serve as key signaling molecules in gut-brain communication; They jointly upregulate tight junction protein expression and promote mucus secretion, effectively repairing the intestinal barrier while indirectly stabilizing the blood-brain barrier to block peripheral pathological factors from invading the central nervous system. They synergistically modulate innate and adaptive immunity, suppressing excessive inflammation and promoting an anti-inflammatory environment, thereby alleviating systemic and neuroinflammation. This multi-targeted, systemic regulatory network aligns closely with PD’s complex, multifactorial pathophysiology. Despite promising prospects, clinical translation faces challenges including: substantial inter-individual gut microbiome heterogeneity, pathological differences across PD subtypes, and the structural complexity of medicinal plant polysaccharides alongside strain-specific probiotic functions. These factors necessitate personalized and precision-oriented synbiotic development. Future research should focus on: leveraging multi-omics technologies and artificial intelligence to identify optimal functional combinations of specific polysaccharide structures with specific probiotic strains; conducting rigorous clinical trials across PD subtypes to validate efficacy and safety; and exploring “tailor-made” synbiotic intervention protocols based on individual gut microbiome profiles.

In summary, integrating the traditional Chinese medicine concept of “medicinal foods” with modern microbiome and neuroscience research, “medicinal plant polysaccharide-probiotic synbiotics” represent a highly promising next-generation nutritional therapeutic strategy capable of multi-pathway intervention in PD pathogenesis.

## Figures and Tables

**Table 1:** Neurotransmitters Produced by Different Probiotic Strains and Their Functions.

Probiotics	Neurotransmitters Produced	Primary Functions Associated with This Neurotransmitter	References
*L. rhamnosus*	GABA	Improves sleep quality and mood, reduces anxiety, depression and stress, regulates behavior	[25]
*L. plantarum*	GABA 5-HT DA Glu Histamine	Improves mood, cognitive performance Reduces anxiety, increases memory Reduces stress Prevents food poisoning	[26,27,28,29]
*L. helveticus*	5-HT NE Glu	Improves depression, anxiety Improves cognitive abilities Regulates gastric reflexes	[30,31,32]
*L. reuteri*	GABA Histamine	Improves psychiatric disorders Modulates immune function	[33,34,35,36]
*B. breve*	GABA	Lowers blood glucose, inhibits foodborne intestinal pathogens, improves obesity	[37,38,39]
*L. acidophilus*	GABA 5-HT	Improves sleep Improves ASD, intestinal inflammation	[40,41,42]
*L. paracasei*	GABA	Improves motor in-coordination, neuroinflammation, sleep duration	[42,43]
*L. casei*	GABA	Relieves ulcerative colitis	[44]
*C. butyricum*	GABA 5-HT	Treats depression Improves learning and memory	[45,46]
*B. subtilis*	GABA 5-HT	Improves sleep, relieves anxiety Regulates intestinal motility	[47,48]

**Table 2:** Motor and Non-Motor Subtypes of Parkinson’s Disease.

Parkinson’s Subtype Classification	Subtype Name	Clinical Characteristics	Pathogenesis	Biomarkers	References
Sport subtype	Tremor-dominant (TD)	Significant resting tremor with milder motor retardation and slower disease progression	Selective preservation of nigrostriatal dopaminergic neurons; overactive cerebello-thalamo-cortical loop	Preserved striatal dopamine transporters; low cerebrospinal fluid alpha-syn levels	[105,106]
	Postural Instability/Gait Impairment Type (PIGD)	Postural instability, frozen gait, high risk of falls, more rapid cognitive decline	Degeneration of cholinergic and non-dopaminergic systems, cortical atrophy	Low cholinergic activity; MRI shows cortical atrophy	[107,108,109]
Non-motorized subtype	Cognitive impairment dominant	Early executive function decline, visuospatial impairment, dementia	Lewy bodies co-deposited with tau protein; abnormal frontal-parietal network connectivity; α-syn Widespread distribution	Elevated CSF tau protein; APOE ε4 allele positive; amyloid PET positive	[110,111,112]
	Autonomic dysfunction type	Upright hypotension, constipation, urinary incontinence	Peripheral autonomic nervous system alpha-syn deposits; cardiac sympathetic denervation	Abnormal cardiac MIBG scintigraphy; decreased CSF norepinephrine levels	[113,114,115]
	Rapid Eye Movement Sleep Behavior Disorder (RBD)	Dreaming enactment behaviors predictive of high risk of alpha-syn disease conversion	Loss of neurons in the pontine pallidum/inferior pontine pallidum; early deposition of α-syn in the brainstem	Elevated cerebrospinal fluid α-syn oligomers; reduced brainstem MRI volume	[116,117]

**Table 3:** Representative clinical studies on probiotic treatment for PD in recent years.

Time	Strains	Intervention Time and Experimental Distribution	Types of Experiments and Detection Indicators	Experimental Effect	References
2016	Fermented milk with probiotics and prebiotics	4 weeks; 120 PD patients; Fermented milk group with multiple probiotic strains and prebiotic fiber (*n* = 80), placebo group (*n* = 40)	Tertiary, randomized, double-blind, placebo-controlled trial; Effect of probiotic group on constipation in patients with PD	containing probiotics and prebiotics resulted in more complete defecation per week than placebo	[118]
2018	*L.acidophilus*, *L. reuteri*, *B. bifidum, L. lactis* (M-SPC)	12 weeks; 50 PD patients; Probiotic Supplement Group 8 × 10^9^ CFU/day (*n* = 25); Placebo group (*n* = 25) (one capsule per day)	Randomized, double-blind, placebo-controlled trials; gene expression of IL-1, IL-8, TNF-α, TGF-β and PPAR-γ	The probiotic group improved the gene expression of IL-1, IL-8, TNF-α, TGF-β, and PPAR-γ, as well as lipid metabolism disorders.	[119]
2021	Multi-Strain Probiotic Capsules	4 weeks; 72 PD patients; probiotic capsule group (*n* = 34) placebo (*n* = 38)	Double-blind, randomized, placebo-controlled, single-center trial; Are Probiotics Effective for Constipation	Probiotic treatment increased the number of weekly bowel movements in the patients	[120]
2021	*L.plantarum* PS128	12 weeks; 25 patients with PD; 6 × 10^11^ CFU/day	Open-label, single-arm, baseline-controlled trial; UPDRS	Improving UPDRS Exercise Scores and Quality of Life in Patients with PD	[121]
2022	*Bifidobacterium animalis lactis subspecies* Probio-M8	3 months; 82 PD patients; Probiotic group (*n* = 48), placebo group (*n* = 34)	Randomized, double-blind, placebo-controlled clinical trials; UPDRS, did non-motor symptoms improve	Probiotics group improves sleep quality, gastrointestinal symptoms, and relieves anxiety; some improvement in motor symptoms	[122]
2024	*B. animalis subsp. lactis* BS01 LMG P- 21384, *B. longum* BL03 DSM 16603, *B. adolescentis* BA02 DSM 18351, oligofructose, maltodextrin groups(A)	12 weeks; Forty patients with PD were randomly assigned in a blinded fashion to one of two groups; >1 × 10^9^ CFU/AFU	Clinical trial method; UPDRS and assessment of the effect of probiotic groups on peripheral cell levels in patients with PD	Compared with the placebo group, group A showed significant improvement in motor and non-motor symptoms, decreased levels of IFN-γ, IL-6, and increased TGF-β.	[99]

**Table 4:** Overview of Plant Polysaccharide Sources, Structural Characteristics, and Bioactive Functions.

Source	Main Glycosidic Linkage/Structural Unit (“→”Indicates the Direction of Glycosidic Bond Linkage)	Main Bioactivity/Mechanism (“↑” Represents Increase, and “↓” Represents Decrease)	References
*Lycium barbarum*	α-1,6-glucan; arabinogalactan; rhamnogalacturonan	Immunomodulation; antioxidant; hepatoprotective; blood glucose regulation; promotes *Lactobacillus* and *Bifidobacterium* ↑	[139,140,141]
*Ziziphus jujuba*	1,3-β-D-glucan; 1,6-β-D-glucan; pectic polysaccharides	Hematopoietic; anti-fatigue; gut microbiota modulation; antioxidant; promotes short-chain fatty acid production	[141,142,143]
*Schisandra chinensis*	→1)-D-Galp-(4→; →1)-D-Glcp-(4→; →1)-D-Marp-(6→	Immunostimulatory; hepatoprotective; antioxidant; modulates gut microbiota (e.g., Bacteroidetes ↑)	[144,145,146]
*Achyranthes bidentata*	1,3-linked Galp; 1,4-linked GalpA; 1,3,5-linked Araf	Anti-inflammatory; osteogenic; immunomodulatory; restructures gut microbiota	[147,148]
*Glycine max*	α-D-Galp-(1→; β-D-Glcp-(1→4)-α-L-Rha-(1→; →4)-α-Galp-(1→2)-α- Rhap-(1→	Hypolipidemic; modulates gut microbiota *(Bifidobacterium* ↑; *Escherichia* ↓); enhances gut barrier	[149,150]
*Panax ginseng*	1,6-α-D-glucan; 1,4-β-D-glucan; arabinogalactan	Immunostimulatory; anti-fatigue; hypoglycemic; antioxidant; modulates gut microbiota (*Lactobacillus* ↑; *pathogens* ↓)	[151,152,153]
*Dioscorea opposita*	1,3-β-D-glucan; 1,6-α-D-glucan; pectic polysaccharides	Blood glucose regulation; improves digestion; immunomodulatory; promotes probiotic growth (e.g., *Lactobacillus*)	[154,155]
*Malus domestica*	(1→3), (1→6)-α/β-glucan; contains Glc, Man, Xyl, Gal units	Antitumor; antioxidant; modulates gut microbiota (*butyrate*-producing bacteria ↑; inflammation-related genera ↓)	[156,157]
*Atractylodes macrocephala*	1,6-β-D-glucan; 1,3-β-D-glucan; pectic polysaccharides	Strengthens spleen and stomach; diuretic; immunomodulatory; modulates gut microbiota (beneficial bacteria ↑; conditional pathogens ↓)	[158,159,160]
*Astragalus membranaceus*	1,4-α-D-Glcp; 1,6-α-D-Glcp; 1,3,6-α-D-Glcp; with minor amounts of arabinose, galactose *	Immunomodulatory; antioxidant; anti-inflammatory; modulates gut microbiota (e.g., *Lactobacillus*, *Bifidobacterium*; ↓)	[161,162]

* Note: ‘→X)-’ denotes a bond attached to carbon X of the sugar; ‘-(Y→’ denotes a bond departing from carbon Y of the sugar. Parentheses are integral to the notation.

## Data Availability

No new data were created or analyzed in this study. Data sharing is not applicable.

## Abbreviations

The following abbreviations are used in this manuscript:

5-HT	5-hydroxytryptamine (serotonin)
ADE	adenine-induced chronic kidney disease
APOE ε4	apolipoprotein E ε4
ASK1	apoptosis signal-regulating kinase 1
BDNF	brain-derived neurotrophic factor
CAT	catalase
CCK	cholecystokinin
CFU	colony-forming unit
CSF	cerebrospinal fluid
CXCR6	C-X-C chemokine receptor type 6
DA	dopamine
DAO	diamine oxidase
DSS	dextran sodium sulfate
EECs	enteroendocrine cells
FXR	farnesoid X receptor
GABA	gamma-aminobutyric acid
Galp	Galactopyranose
GDNF	glial cell line-derived neurotrophic factor
Glcp	Glucopyranose
GLP-1	glucagon-like peptide-1
GM-CSF	granulocyte-macrophage colony-stimulating factor
GPNE-I	ginseng polysaccharide NE-I
GPR41/43	G protein-coupled receptor 41/43
GSH	glutathione
GSH-Px	glutathione peroxidase
HDAC6	histone deacetylase 6
HPA axis	hypothalamic-pituitary-adrenal axis
IFNγ	interferon-gamma
IgA	immunoglobulin A
IL-1β, IL-6, IL-10	interleukin-1β, -6, -10
IL-2, IL-17	interleukin-2, -17
ISAPP	International Scientific Association for Probiotics and Prebiotics
JNK	c-Jun N-terminal kinase
Keap1	Kelch-like ECH-associated protein 1
LPS	lipopolysaccharide
Lrp5	low-density lipoprotein receptor-related protein 5
MDA	malondialdehyde
MGBA	microbiota-gut-brain axis
MIBG	meta-iodobenzylguanidine
MPTP	1-methyl-4-phenyl-1,2,3,6-tetrahydropyridine
MRI	magnetic resonance imaging
NE	norepinephrine
NF-κB	nuclear factor kappa B
NK cells	natural killer cells
NLRP3	NLR family pyrin domain containing 3
NMDA	N-methyl-D-aspartate
Nrf2	nuclear factor erythroid 2–related factor 2
NTS	nucleus tractus solitarius
p38 MAPK	p38 mitogen-activated protein kinase
PD	Parkinson’s disease
PET	positron emission tomography
PIGD	postural instability/gait difficulty
PYY	Peptide YY
RBD	rapid eye movement sleep behavior disorder
ROS	reactive oxygen species
SCFAs	short-chain fatty acids
SIgA	secretory immunoglobulin A
SLAMF6	signaling lymphocytic activation molecule family member 6
SOD	superoxide dismutase
STAT1/6	signal transducer and activator of transcription 1/6
TD	tremor-dominant
TGF-β	transforming growth factor-beta
Th17	T helper 17 cells
TLR4	Toll-like receptor 4
TNF-α	tumor necrosis factor-alpha
TRAIL-DR5	TNF-related apoptosis-inducing ligand—Death receptor 5
Tregs	regulatory T cells
TXN	thioredoxin
UPDRS	Unified Parkinson’s Disease Rating Scale
VH/CD	villus height/crypt depth
ZO-1	zonula occludens-1
α-syn	alpha-synuclein
